# The mechanism of pseudouridine synthases from a covalent complex with RNA, and alternate specificity for U2605 versus U2604 between close homologs

**DOI:** 10.1093/nar/gkt1050

**Published:** 2013-11-07

**Authors:** Nadine Czudnochowski, Gary W. Ashley, Daniel V. Santi, Akram Alian, Janet Finer-Moore, Robert M. Stroud

**Affiliations:** ^1^Department of Biochemistry and Biophysics, University of California, San Francisco, 600 16th Street, San Francisco, CA 94158, USA, ^2^ProLynx, 455 Mission Bay Blvd., Suite 145, San Francisco, CA 94158, USA and ^3^Faculty of Biology, Technion-Israel Institute of Technology, Technion City, Haifa 320003, Israel

## Abstract

RluB catalyses the modification of U2605 to pseudouridine (Ψ) in a stem-loop at the peptidyl transferase center of *Escherichia coli* 23S rRNA. The homolog RluF is specific to the adjacent nucleotide in the stem, U2604. The 1.3 Å resolution crystal structure of the complex between the catalytic domain of RluB and the isolated substrate stem-loop, in which the target uridine is substituted by 5-fluorouridine (5-FU), reveals a covalent bond between the isomerized target base and tyrosine 140. The structure is compared with the catalytic domain alone determined at 2.5 Å resolution. The RluB-bound stem-loop has essentially the same secondary structure as in the ribosome, with a bulge at A2602, but with 5-FU2605 flipped into the active site. We showed earlier that RluF induced a frame-shift of the RNA, moving A2602 into the stem and translating its target, U2604, into the active site. A hydrogen-bonding network stabilizes the bulge in the RluB–RNA but is not conserved in RluF and so RluF cannot stabilize the bulge. On the basis of the covalent bond between enzyme and isomerized 5-FU we propose a Michael addition mechanism for pseudouridine formation that is consistent with all experimental data.

## INTRODUCTION

In all kingdoms of life non-coding RNAs are extensively post-transcriptionally modified. Approximately 0.8% of the total coding capacity of *Escherichia coli (E. coli)* is devoted to RNA modifying enzymes, underscoring the biological importance of RNA modification (1,2). Modifications cluster around functionally important sites, for example the peptidyl transferase center of the ribosome and the tRNA anticodon stem-loop, where they contribute to the efficiency and fidelity of mRNA translation (3,4).

Isomerization of uridine to its *C*-glycoside isomer, pseudouridine (Ψ), is the most prevalent RNA modification (4). The minimal mechanism for this reaction involves cleavage of the *N*-glycosidic bond of the target residue, rotation of the cleaved uracil to juxtapose C5 of the pyrimidine and C1′ of the ribosyl moiety of RNA, and formation of the C1′-C5 carbon–carbon bond. An Asp that is conserved in all known pseudouridine synthases (Ψ synthases) has been implicated in catalysis (5), although its role is still debated (6,7). In our favored mechanism it catalyses the reaction by Michael addition to C6 of the base and in an alternate proposal, the acylal mechanism, it adds to the C1′ of the ribose to displace the uracil (Scheme 1) (5).
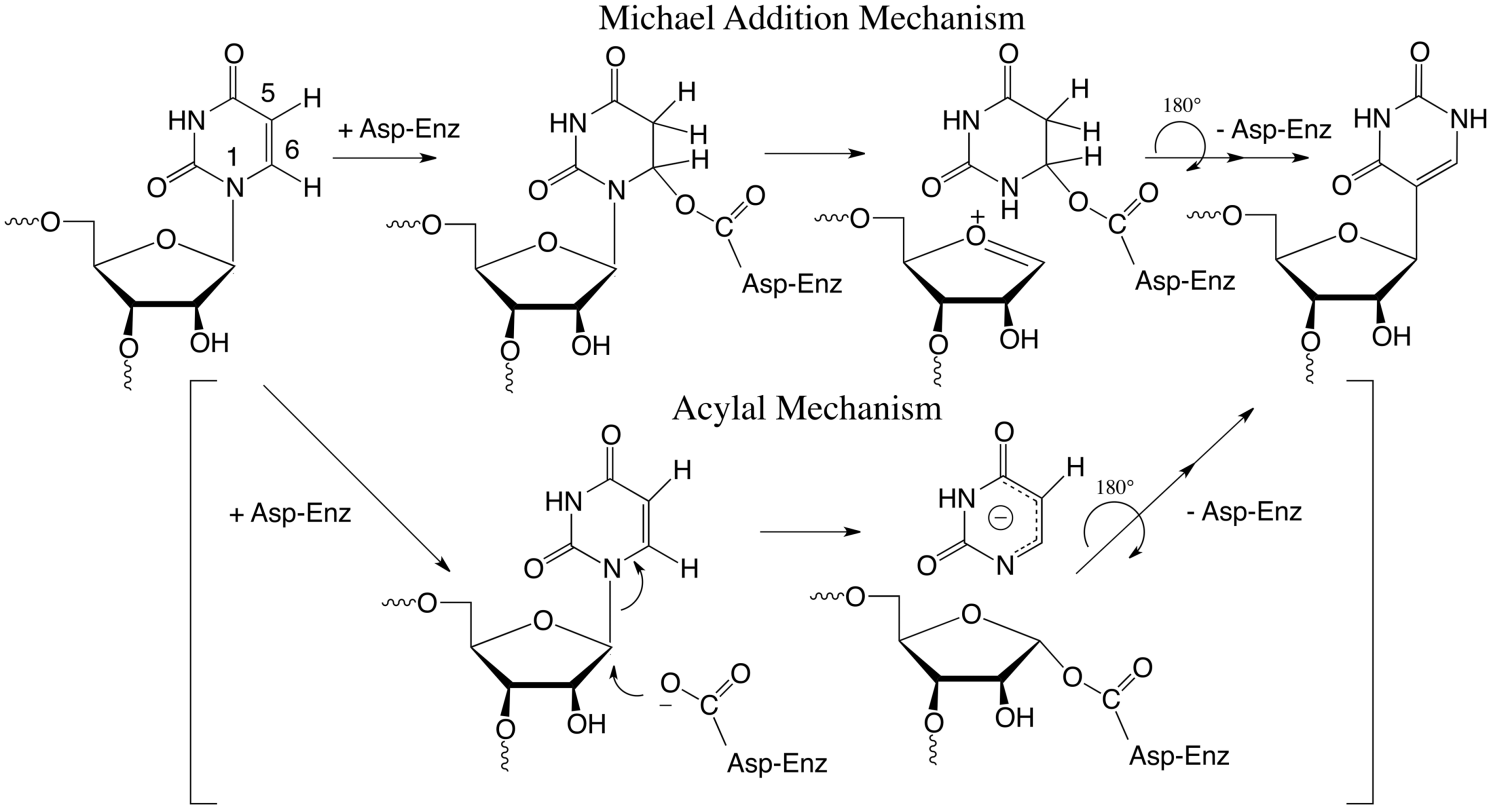

**Scheme 1.** Proposed mechanisms for Ψ synthases.

Ψ synthases use two strategies for selecting a target for modification. In eukaryotes and archaea, pseudouridylations of rRNA and snRNAs are accomplished with versatile ribonucleoprotein particles (RNPs) comprising the Ψ synthase Cbf5 (dyskerin in humans), three structural proteins and a small guide RNA, which is responsible for target recognition by base pairing to either side of the target site (8).

All kingdoms of life also have stand-alone Ψ synthases that modify sites that may be buried in folded RNA, thus cannot be recognized by direct sequence readout (8). Some of the Ψ synthases have multiple substrates, including non-coding RNAs implicated in control of gene expression and mRNA translation. Insights into how stand-alone Ψ synthases recognize their targets have largely come from crystal structures of bacterial Ψ synthases in complexes with substrate RNAs. These crystal structures revealed that conformational flexibilities of both the enzyme and its RNA substrate were critical for target specificity (9–14).

Here we address the question of how two homologous *E. coli* Ψ synthases, RluB and RluF, selectively modify adjacent bases on a stem-loop of *E. coli* 23S rRNA. RluB is specific for U2605 whereas RluF is selective for U2604, but also modifies U2605 to a small extent (15). The basis for the different target selectivities is not immediately clear from the sequences, which are ∼31% identical. The catalytic cores of Ψ synthases are often decorated with inserts or extra domains that contribute to substrate recognition and target specificity (16). The domain structures of RluB and RluF are the same and there are no unique inserts of more than four residues that could explain the differences in specificity.

We determined the structure of *E. coli* RluF in a complex with a 22-mer RNA substrate analog identical in sequence to the substrate rRNA stem-loop, except with the target U2604 substituted by 5-fluorouridine (5-FU) to block a late step in catalysis (11). The structure showed that association with RluF induces a rearrangement of the RNA stem-loop, resulting in a frame-shift in base pairing. A bulge in the RNA is induced to fold into the stem, causing the RNA 3′ to the bulge to translate by 1 nt, thereby flipping out U2604 into the active site. We surmised that the same RNA stem-loop would not rearrange upon binding to RluB, leaving U2605 positioned for flipping into the active site. We now present the 1.3 Å structure of RluB in complex with a 21-mer stem-loop substrate, in which U2605 is substituted by 5-FU. The structure provides a rationale for the different specificities of RluF and RluB. It also reveals a covalent bond between the phenolic hydroxyl of the conserved active site Tyr140 and C6 of the isomerized 5-FU. The covalent bond between Tyr140 and isomerized U2605 occurs by a mechanism similar to that proposed by Gu *et al.* (6), and together with other previous information provides a unified mechanism for Ψ synthases.

## MATERIALS AND METHODS

### Protein expression and purification

A C-terminal truncation of *E. coli* RluB (residues 1–251) was cloned into a modified pET47 vector using the restriction sites BamHI/XhoI to yield a fusion protein with an N-terminal hexahistidine tag and an HRV 3C protease site. RluB (1–251) was expressed in *E. coli* BL21(DE3) cells at 20°C for 16 h after induction with 0.3 mM IPTG. Cells were harvested, washed in Tris-buffered saline and resuspended in lysis buffer (50 mM Hepes pH 7.5, 500 mM NaCl, 5 mM β-mercaptoethanol). The cells were lysed using an Emulsiflex-C5 homogenizer (Avestin) and the lysate was cleared by centrifugation at 32 000 g for 25 min. The supernatant was incubated with nickel-nitrilotriacetic acid (Ni-NTA) at 4°C in the presence of 20 mM imidazole. The resin was washed with 15 column volumes of lysis buffer containing 20 mM imidazole and the fusion protein was eluted with lysis buffer containing 250 mM imidazole. Proteolytic removal of the hexahistidine tag using HRV 3C protease was performed during dialysis of the protein over night at 4°C in 50 mM Hepes pH 7.5, 300 mM NaCl and 0.5 mM TCEP. The cleaved hexahistidine tag was removed by immobilized-metal affinity chromatography using Talon resin (Clonetech). The protein was concentrated using Amicon centrifugal filter units, loaded on a Superdex S200 (10/30) size exclusion column (GE Healthcare) equilibrated in 50 mM Hepes pH 7.5, 300 mM NaCl and 0.5 mM TCEP and eluted with the same buffer. Peak fractions were analysed by SDS-PAGE, concentrated to 10 mg/ml and flash-frozen in liquid nitrogen. RluB mutants were expressed and purified as described for the wild-type protein.

### Crystallization of apo-RluB and the RluB–RNA complex

We were unable to crystallize full-length RluB (291 residues) either alone or with small RNA substrates; therefore we used a construct (residues 1–251) lacking the C-terminal 40 residues, which are predicted to be disordered, for crystallization. Crystals of RluB (1–251) were grown at a concentration of 10 mg/ml in 0.1 M tri-sodium citrate pH 5.6, 20% (v/v) isopropanol and 15% (w/v) PEG4000 using hanging drop vapor diffusion by mixing 1 µl protein with 1 µl reservoir solution (500 µl reservoir solution). To make a heavy atom derivative for determining X-ray diffraction phases RluB crystals were soaked with K_2_PtCl_4_ (4 mM final concentration) for 30–45 min, washed two to three times in reservoir solution and cryoprotected in reservoir solution containing 20% (v/v) ethylene glycol.

The 21-mer stem-loop used for crystallization was identical in sequence with nucleotides 2587–2607 of the *E. coli* 23S rRNA, but with 5-FU substituted for U2605. RNA was purchased from Dharmacon (Thermo Scientific) and deprotected according to the manufacturer’s protocol. The RluB-RNA complex was formed by incubating RluB at a concentration of 5 mg/ml with a 1.2-fold molar excess of 21-mer RNA for 1 h at room temperature. Crystals were obtained in 16% (v/v) polypropylene glycol 400 and 12% (v/v) 1-propanol using hanging drop vapor diffusion. Diffraction data were collected on beam line 8.3.1 of the Advanced Light Source (Berkeley, USA).

### Structure determination

Diffraction data were processed with XDS (17). The native apo-RluB crystals were twinned; however the K_2_PtCl_4_-soaked crystals of apo-RluB were not. The structure of the Pt derivative was therefore solved by molecular replacement using the RluF structure (PDB ID: 2GML) edited with Sculptor as a search model, using Phaser (18). The initial model was fit to a map that had been obtained by experimental phasing of data collected at λ = 1.068830 Å (remote from the Pt absorption peak) using Phenix AutoSol (19). Refinement against the Pt derivative data collected at λ = 1.068830 Å was performed with Phenix.refine (20) and included TLS (21) refinement (2 groups). COOT (22) was used for model building and visualization. Two Pt^2+^ and one Cl^−^ were located in the structure, both at the enzyme surface. One of the Pt^2+^ ions was at a crystal interface, where it regularized packing of RluB molecules in the crystal, thereby resolving the twinning. A correction for anomalous scattering by the Pt was applied during refinement. The RluB–RNA complex structure was solved by molecular replacement with Phaser. A search model was constructed that included the catalytic domain of apo-RluB, the S4 domain of RluF and the ribosomal RNA stem-loop encompassing nucleotides A2590–G2607 without A2602. Refinement was performed with Phenix.refine and included anisotropic ADP refinement in the final cycle. We did not include NCS restraints in our refinement strategy. Protein, RNA and water molecules in the asymmetric unit were refined independently. Data collection and refinement statistics are shown in Table 1. *R*_meas_ represents the redundancy-independent *R*-factor as described in Diederichs and Karplus (23). Electrostatic potentials were calculated with APBS (24). All molecular presentations were prepared with PyMOL (25).

**Table 1.** Data collection and refinement statistics

	RluB–RNA	apo-RluB
Data collection		
X-ray source	ALS 8.3.1	ALS 8.3.1
X-ray wavelength (Å)	1.115869	1.068830
Space group	C2	P4_3_2_1_2
Cell dimensions		
* a*, *b*, *c* (Å)	80.9, 42.2, 169.4	82.4, 82.4, 74.9
β (°)	102.6	
Resolution (Å)^a^	50-1.3	58-2.5
	(1.35-1.3)	(2.6-2.5)
No. of observed/unique reflections	309 954/126 732	176 589/9324
*R*_meas_ (%)^b^	5.5 (108.9)	10.2 (157.5)
*I*/σ(*I*)	13.5 (1.1)	21.5 (2.6)
Completeness (%)	92.0 (85.1)	99.4 (94.2)
Redundancy	2.4 (2.2)	18.9 (17.5)
Refinement		
Resolution	40.5-1.3	58-2.5
*R*_work_/*R*_free_ (%)	17.3/20.3	23.1/26.1
No. of water molecules	630	19
Average *B*-factors (Å^2^)		
Protein, chain A	20	70
Protein, chain B	44	
RNA, chain A	15	
RNA, chain B	41	
Water	32	53
rms deviations		
Bond lengths (Å)	0.009	0.006
Bond angles (°)	1.309	0.917
Ramachandran Plot (%)		
Favored regions	98.6	96.8
Allowed regions	1.2	2.7
PDB ID	4LGT	4LAB

^a^Values in parentheses refer to the highest resolution shell.

^b^Redundancy-independent *R*-factor (on intensities) (23). As given by XDS (17).

### RNA synthesis and tritium release assays

The 21-mer RluB stem-loop was *in vitro* transcribed using the MEGAshortscript T7 Kit (Ambion) and an oligodeoxynucleotide template in the presence of 0.3 mM cold UTP, 0.1 mM [5-^3^H]-UTP (20.6 Ci/mmol, Moravek Biochemicals) and 3.75 mM ATP, GTP and CTP for 3 h at 37°C. The reaction was treated with DNAse I, RNA was extracted with phenol/chloroform and EtOH precipitated. The RNA was further purified by DEAE Sepharose chromatography (GE Healthcare) using a NaCl gradient. RNA containing fractions were collected at 0.4 M and 0.6 M NaCl. RNA was EtOH precipitated and resuspended in water. Activity assays were carried out at room temperature in 50 mM Hepes pH 7.5, 50 mM NaCl and 1 mM TCEP in a reaction containing 50 nM RluB and 0.5 µM RNA. After 1 h the reaction was quenched with 5% (w/v) activated charcoal (Norit A) in 0.1 N HCl, the sample was centrifuged (5 min, 5000 g) and the supernatant was again treated with Norit A, followed by centrifugation. The supernatant was filtered through Ultrafree-MC centrifugal filters (Millipore) to remove residual Norit A. The filtrate was mixed with Aquasol-2 (Perkin Elmer) and released ^3^H was counted. RluB Y140F had 2.8% ± 0.7% activity (mean of five independent measurements) and RluB R108A had 0.6% ± 1.4% activity of wild-type RluB (mean of three independent measurements).

## RESULTS

### The RluB–RNA complex at 1.3 Å resolution

**Figure 1.**
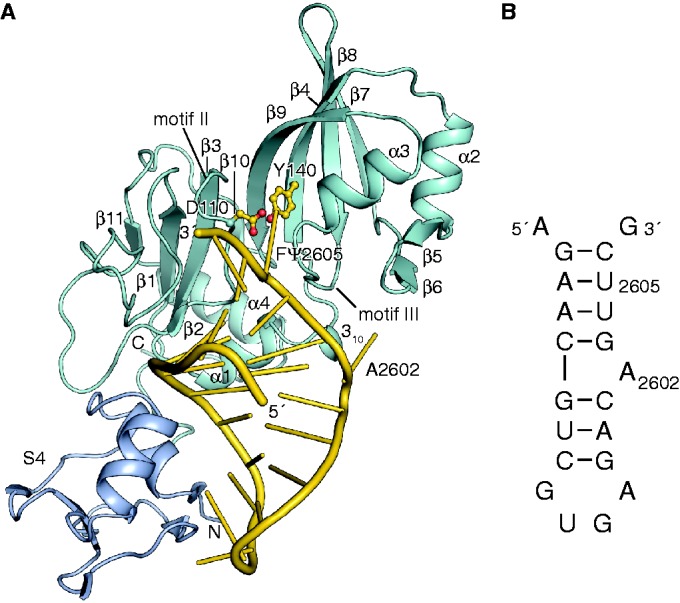
Overall structure of the RluB-RNA complex. (**A**) Structure of RluB-bound to a small RNA substrate. The S4 domain is blue, the catalytic domain is blue–green and the RNA phosphate backbone is gold. Nucleosides are shown as gold sticks. The catalytic aspartate 110 and tyrosine 140 are shown in ball-and-stick form with side chains colored gold. (**B**) Schematic drawing of the 21-mer rRNA small substrate of RluB. For crystallization U2605 was substituted by 5-FU.

*Escherichia coli* RluB (1–251) was co-crystallized with a 21-mer RNA stem-loop identical in sequence to the target stem-loop in 23S rRNA except that U2605 was modified to 5-FU to block the last step in catalysis (Figure 1B). The 40 deleted C-terminal residues in the RluB construct are homologous to the C-terminal domain of *E. coli* RluF, and in an RluF–stem-loop complex the C-terminal domain is completely disordered indicating it does not contribute to binding of the stem-loop (11,26). The truncated C-terminal residues are poorly conserved among RluBs from different species. Thus comparison of the RluF and RluB (1–251)-stem-loop complexes should reveal the salient RluB–RNA interactions responsible for their different specificities.

Crystals of this complex in space group C2 contained two independent RluB–RNA complexes per asymmetric unit, which are essentially the same with an rmsd of 0.9 Å. The unit cell is tightly packed, with an estimated solvent content of 37%, and the main conformational differences between the two complexes are in loops on the periphery of the protein that are involved in crystal contacts. The unique aspect of the active site is a covalent link between a tyrosine and the target base in one of the complexes, to be discussed below. At 1.3 Å resolution, the highest resolution structure of a Ψ synthase/RNA complex to date, the RNA–protein interactions are accurately described.

RluB (1–251) is comprised of an N-terminal S4 domain (27) (residues 1–60) connected by a flexible linker to a catalytic domain (residues 66–251). The S4 domain is conserved in sequence and structure to the S4 domains of RluF and RsuA (11,28). The catalytic domain adopts a mixed α/β-fold that is common to all Ψ synthases (Figure 1A). It consists of an antiparallel eight-stranded bifurcated beta sheet that is flanked by loops, two short beta strands and helices on one face of the sheet. The active site cleft of the enzyme is located in the center of this ß-sheet. The two central strands of the β-sheet, ß3 and ß9, form the floor of the cleft and two of the conserved motifs characteristic of all Ψ synthases (motifs II and III) form the cleft walls (29,30). Motifs II and III contain conserved residues implicated in substrate binding, including the catalytic Asp110 and Arg194.

The 21-mer RNA stem-loop binds with the target base 5-FU2605 flipped out into the active site cleft and the loop region abutting the S4 domain. The stem-loop has the same secondary structure as it has in the context of the large ribosomal subunit, with A2602 forming a bulge (31). In contrast, the base pairing of the same stem-loop rearranges upon binding to RluF (11). In the RluF–stem-loop complex A2602 was refolded into the stem and nucleotides 3′ to A2602 had translated one position to place U2604 at the active site.

### Trapping with 5-FU2605 reveals a conserved tyrosine rather than water bound to C6

**Figure 2.**
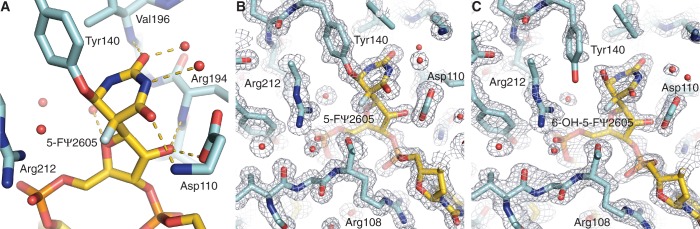
Active site of the RluB-RNA complex. (**A**) View of the active site of the first molecule with protein and RNA shown as sticks and H-bonding interactions of the target base and ribose with neighboring protein residues and water depicted as gold dashes. (**B**) and (**C**) 2Fo-Fc (α_calc_) density at the active sites of the two molecules in the asymmetric unit of RluB–RNA crystals, contoured at 1.5 σ and 1.2 σ, respectively. (B) The density map for the first molecule clearly shows a covalent bond between conserved Tyr140 and the isomerized target base. Asp110 is the catalytic Asp. (C) Tyr140 in the second molecule is in partial density, indicating this molecule may exist as a mixture of covalent and non-covalent RNA complexes in the crystal.

The target nucleotide, U2605, is flipped into the active site of the enzyme and RluB has turned over 5-FU2605 to give rise to the C1′-C5 glycoside bond typical for Ψ; RluB (1–251) is catalytically active against the 21-mer (Figure 2A). The 5-fluoro substituent cannot be abstracted to generate the product thus C5 is tetrahedral and the nucleotide is in a bent configuration with the pyrimidine ring tilted ∼120° from the plane of the ribose.

In crystal structures of other Ψ synthase complexes with 5-FU substituted substrates, the 5-FU has both isomerized and been hydrated at C6 to give (5S, 6R) 5-fluoro-6-hydroxy-Ψ (**8** in Scheme 4) (9,11–14). But in the RluB structure, one of the RluB–RNA active sites shows clearly that Tyr140, perhaps fortuitously, forms a covalent bond with C6 of the isomerized base (Figure 2A and B). The Tyr140 hydroxyl added cis to the fluoro substituent, just as water did in the hydrated product thus giving rise to the same stereochemistry at C6. It is positioned on the opposite side of the pyrimidine plane than the universally conserved catalytic Asp110. This RluB–RNA structure is the first Ψ synthase structure with a covalent bond between enzyme and its RNA substrate.

The second RluB–RNA complex is statistically disordered at this site indicating a mixture of covalent and non-covalent complexes (**8** in Scheme 4) (Figure 2C). In other Ψ synthase–RNA complexes the backbone is very similar though the tyrosine hydroxyl points away from C6 toward the phosphate moiety of the target and there are small compensating differences in the catalytic aspartate.

Tyr140, though highly conserved, is not invariant among Ψ synthases (32). We mutated Tyr140 to Phe in RluB (1–251) and measured enzyme activity by a tritium release assay to be ∼3% of wild-type RluB (1–251) after an incubation time of 60 min. Thus Tyr140 contributes to catalysis, perhaps by providing binding stability or orienting the target base, but it is not an essential catalytic residue. This result is consistent with the results of mutating the Tyr in other Ψ synthases. Several Tyr mutants in human Pus1 are partially active (33). A crystal structure of the Phe variant of TruB in complex with a 5-FU-substituted RNA substrate showed the 5-FU had converted to 6-OH-5-FΨ, thus the mutant was competent to undergo the first steps in catalysis (34).

### Substrate specificity and arginine-induced base flip-out from the RNA stem

**Figure 3.**
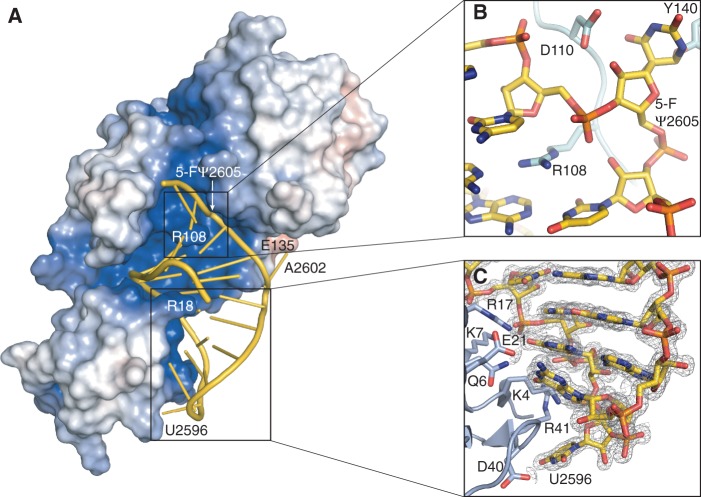
Interactions between RluB and the 21-mer RNA stem-loop. (**A**) Surface representation of RluB colored according to its electrostatic surface potential with positive electrostatic potential in blue (+7 kT/e) and negative potential in red (–7 kT/e). The 21-mer RNA is represented in gold. The orientation of RluB is as in Figure 1A. (**B**) Arginine 108 intercalates into the RNA stem. RNA and protein residues are shown as gold and blue–green sticks, respectively. (**C**) The S4 domain of RluB anchors the loop of the RNA to the protein. RluB is shown in blue, side chains interacting with the RNA (colored gold) are depicted as sticks. The 2Fo-Fc density for the RNA is contoured at 1.5 σ.

The 21-mer stem-loop binds to an electropositive-binding groove, bounded on either side by conserved Ψ synthase motifs that form the walls of the catalytic cleft (Figures 1A and 3A). Motif II, which contains catalytic Asp110, binds the minor groove. Motif III binds the major groove of the RNA. Helix α1 and the ‘forefinger loop’ following β1 also bind to the minor groove, as in the RNA complexes of RluF and RluA (11,14). Another common determinant of RNA recognition, the ‘thumb loop’ (between α2 and β7) is too short to interact with the stem-loop RNA in RluB (9,14). The S4 domain binds the major groove of the loop end of the RNA, thus pinning the stem-loop at one end and fixing the alignment of U2605 with the active site. Figure 3C shows S4 domain residues involved in hydrogen-bonding interactions with loop and adjacent stem nucleotides.

**Figure 4.**
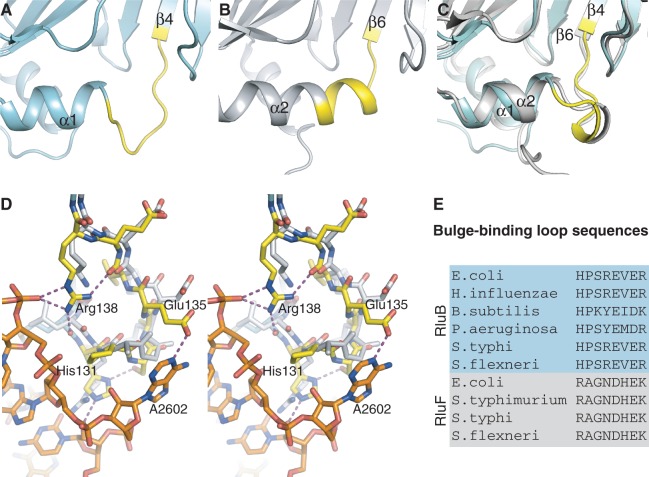
Comparison of the bulge-binding loop in RluB and RluF. (**A**) apo-RluB is colored blue–green and the bulge-binding loop (residues 131–138) is colored yellow. (**B**) apo-RluF in gray with analogous residues (128–135) colored yellow. (**C**) Superposed RluB and RluF in their RNA-bound conformations (RNA omitted for clarity). RluB is blue–green with its bulge-binding loop highlighted in yellow. RluF is gray. The loops have refolded to the same structure in the two RNA-bound complexes. (**D**) Stereo plot of the superposed RluB– and RluF–RNA complexes in the vicinity of the bulge (A2602). RluB is plotted with blue–green carbons and RluF with gray carbons. The bulge-binding loop in RluB is shown with yellow carbons and hydrogen bonds from this loop are shown as purple-dashed lines. (**E**) Aligned sequences of loop residues from several species of RluB and RluF.

The bound 21-mer RNA adopts the same secondary structure as in the ribosome, with a stem consisting of seven Watson–Crick base pairs, a 4-nt loop, and a bulge formed by A2602 (PDB ID: 2I2T, helix H69) (31), though the backbone is more extended around A2602 and G2603. A similar, albeit less dramatic extension of the stem-loop backbone was present in the RluF–RNA structure. A2602 in the RluB–RNA structure packs against the loop connecting α1–β4, making van der Waals contacts with Pro132 and Ser133 and a hydrogen bond between N1 and the carboxyl of Glu135 (Figure 4D).

**Figure 5.**
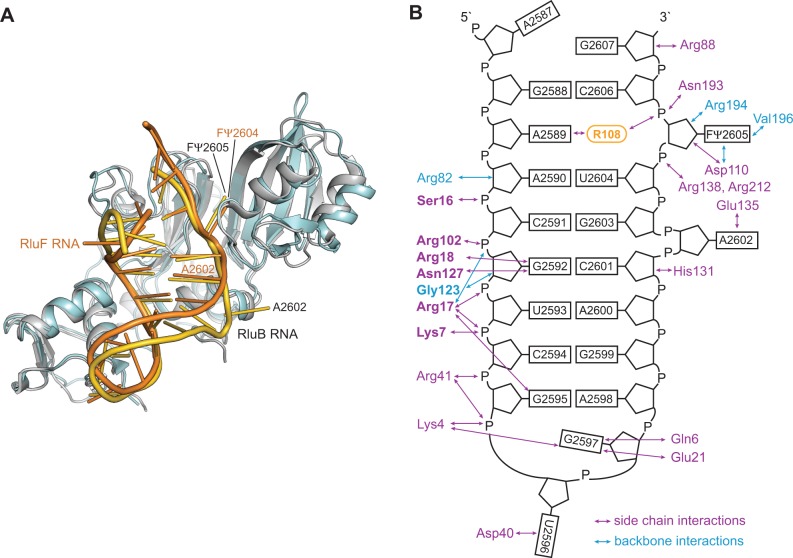
RNA recognition by RluB and RluF. (**A**) Comparison of RluB–RNA (blue–green and gold) that targets U2605 and RluF–RNA (gray and orange) that targets U2604. In the latter A2602 is refolded into the stem, base registry is shifted up by one base on the site 3′ of A2602, and U2604 is flipped out (11). The RNAs overlay closely in the RNA-binding grooves of the catalytic domains. (**B**) Representation of hydrogen bond interactions between residues of RluB and nucleotides of the RNA stem-loop. Residues that are conserved in RluF and engage in interactions with the same nucleotide (from A2590 to U2596) as RluB are highlighted in bold.

RluB–RNA interactions generally parallel those seen in the RluF–stem-loop complex (11). The side chain of Arg17 plays a major role in anchoring the RNA to the S4 domain, forming direct hydrogen bonds with the phosphates of U2593 and C2594 and with the base of G2595. In addition its backbone amide hydrogen bonds with the phosphates of G2592 (Figures 3C and 5B). Arg108 intercalates into the stem where U2605 is flipped into the active site, and stacks between base pairs G2588-C2606 and A2590-U2604 (Figure 3B).

Arg108 is conserved in the RluA, RsuA and TruA families and using MD simulation we demonstrated how this residue might assist in guiding the target base into the active site of the enzyme TruA (10). In an RluA–RNA complex, as in our RluB–RNA structure, the homologous Arg substitutes for the flipped-out target base in the bound RNA stem-loop (14). Variants of RluA where a Met or Lys is substituted for this Arg are inactive, suggesting the Arg has an essential role in assisting base-flipping or stabilizing the flipped-out conformation (14). We mutated Arg108 to Ala and the mutant had essentially zero (0.6% of wild-type) activity, consistent with the RluA mutagenesis result. In members of the TruB family a histidine residue assists in nucleotide flipping (9).

Many of the hydrogen bond interactions between the RNA and RluB are mediated by water. We identified 30 waters in the RNA–RluB interface of complex A (the better ordered of the two complexes in the asymmetric unit), 18 of which were also seen in complex B. The water-mediated nature of much of the interface makes it plastic, allowing RluB and RluF to bind the same stem-loop substrate in native and rearranged forms, respectively, using many analogous interactions. Three of the interface waters are at the active site and these may have important roles in orienting the target base during catalysis or for catalysis itself.

### Comparison between apo-RluB and apo-RluF

Substrate binding by Ψ synthases involves conformational changes to both protein and RNA thus the structures of the apo enzyme and any intermediates along the binding trajectory are important for substrate specificity (10). The crystal structure of apo-RluB (1–251) was therefore solved to a resolution of 2.5 Å with one RluB molecule per asymmetric unit. The S4 domain (residues 1–60) is not visible in the structure of apo-RluB indicating that this domain is either disordered or flexible in the absence of RNA; flexibility between the S4 domain and the catalytic core has also been reported for the Ψ synthases RsuA and RluD (28,35,36).

Apo-RluB is similar to apo-RluF (26) with an rmsd of 1.9 Å (over 164 Cα) with a highly conserved core that encompasses the central beta sheet and helices α1–α3 (rmsd of 1.1 Å over 126 residues). Differences are localized to insertions of 1–4 residues in non-conserved loops on the periphery of the proteins, however there are two significant differences between the structures relevant to the different substrate specificities of RluF and RluB. First, the residues that form two C-terminal turns of helix α2 in RluF form a loop in RluB (Figure 4A and B). These residues interface with the bulge region of the substrate in the RluB–RNA complex. Second, in RluB, helix α4 is followed by a hairpin turn that changes the direction of the peptide chain and allows it to pack against α4 instead of impinging on the bulge-binding site. In apo-RluF the residues C-terminal to the helix do not reverse direction and do not have a regular secondary structure. This difference persists in the RNA-bound complexes of the enzymes (Supplementary Figure S1).

### Conformational changes of RluB upon RNA binding

There are three major conformational changes to RluB that occur upon RNA binding. First, the S4 domain becomes well ordered in presence of RNA and binds to the major groove and the loop region of the RNA. Secondly, RluB undergoes a rigid body hinge motion of two subdomains around the active site cleft upon RNA binding. Superposition of the apo-RluB and the RNA-bound RluB structures results in a large rmsd of 2.1 Å (over 185 Cαs), however individually the subdomains align closely. Alignment of both subdomains, allowing for flexibility in their relative orientations (‘flexible alignment’ in the program RAPIDO (37)), results in a low rmsd of 0.5 Å (over 163 Cαs). Superposition on either subdomain illustrates the closing of the rigid bodies around the RNA; the hinge axis goes through the beta sheet at the active site of the enzyme. Similar hinge motions around the active site cleft have been seen for RluF and TruB (11,38). Finally, residues 132–134 in the loop between α1 and β4 refold into a 3_10_ helix and bind to the RNA near the bulge (Figure 4C).

### Favoring the bulge in RluB but not in RluF

The stem-loop binds to RluB with the same secondary structure as it has in the ribosome. The bulge at A2602 binds underneath α1 with its purine and ribose moieties packed against the 3_10_ helix following α1 (Figure 4C and D). RluB stabilizes the bulge with a repertoire of hydrogen bonds involving side chains of His131, Ser133, Glu135 and Arg138, and flips out U2605 into the active site. Glu135 accepts a hydrogen bond from N1 of A2602 and is the only residue in hydrogen-bonding contact with the bulge nucleotide. Arg138 tethers the bulge-binding loop to the stem-loop by donating hydrogen bonds to both the RNA and protein backbone (Figure 4D). In addition several water molecules engage in hydrogen-bonding interactions with residues 131–138 and the RNA and extend the hydrogen-bonding network.

In RluF, residues equivalent to RluB residues 131–138 achieve the same protein fold as in RluB upon RNA binding (Figure 4B and C), but the different side chains of these residues cannot stabilize the bulge in the RluF complex (Figure 4D). For example, the RluF residue homologous to Glu135 is a conserved Asp, which is too short to reach the bulge. Residues 131–138 are highly conserved among RluBs from different species, and the corresponding residues in RluF are conserved among RluF species, suggesting the residues are a distinguishing feature of the enzymes related to their different specificities (Figure 4E).

When the stem-loop binds to RluF, the bulge at A2602-folds into the stem, the RNA bases 3′ to A2602 are frame-shifted, and U2604 flips out into the active site. Nucleotides from G2597 to A2602, which are not in contact with the protein, are shifted by 3–4 Å relative to their positions in the RluB complex because of the different geometry at A2602 (Figure 5A). RluB and RluF make congruent hydrogen-bonding interactions with nucleotides from A2590 through U2596 on the other strand of the stem-loop (Figure 5B).

## DISCUSSION

### Structural basis for alternate specificity to adjacent bases

RluB-bound to the isolated ribosomal stem-loop, nt 2587–2607, with 5-FU2605 substituted for the target, shows that Arg108 displaced 5-FU2605 from the stem into the active site (just as the homologous Arg in RluF displaced 5-FU2604), where it went through the initial steps of isomerization to pseudouridine. Why does the stem-loop rearrange upon binding to RluF but not to RluB? While the RNA-binding cores of the two enzymes are highly conserved, and their binding interactions with one strand of the RNA stem (A2590- U2596) are equivalent, in RluF the bulge at A2602 is pushed back into the stem and the base pairing is frame-shifted, allowing U2604 instead of U2605 to flip into the active site (Figure 5A). Seeking the basis for compression of the bulge and concomitant frame-shift in RluF we aligned the substrate RNA from the RluB complex onto the RluF–RNA complex structure. The last ordered stretch of residues in RluF, immediately prior to the apparently disordered ∼40 amino acids at the C-terminus, make different interactions with the aligned RNA than the equivalent residues in RluB make in the RluB–RNA complex (Supplementary Figure S1); however, since this stretch is probably flexible and not part of the catalytic core it is unlikely to drive stem-loop rearrangement.

More likely, stabilizing interactions between the bulge and residues 131–138 connecting α1 and β4 (RluB nomenclature) prevent stem-loop rearrangement in RluB, while absence of such interactions allow rearrangement in RluF. Residues 131–138 are different between RluBs and RluFs but highly conserved among different species of each enzyme. Specifically, Glu135 (conserved in RluBs) makes a hydrogen bond to the bulge base A2602, while the equivalent Asp, (conserved in RluFs) is too short to make such a contact.

In the apo-RluB structure residues 131–138 form a loop, whereas in the apo-RluF structure these residues are a two-turn extension of a helix (α2 in RluF) that directly overlaps the bulge-binding site. The apo-conformation of residues 131–138 might be part of the basis for antagonizing the bulge in RluF. While the frame-shifted state of RNA in the RluF–RNA complex leads to catalysis at U2604, the energy difference favoring frame-shifted over unrearranged stem-loop in the RluF–RNA complex must be rather small since RluF does modify the RluB target, U2605, to a small extent *in vivo* (15), and is slightly active against stem-loop mutants with a uridine at 2605 and not at 2604 (11).

### The Ψ synthase reaction mechanism

Our RluB-stem-loop structure is the first crystal structure of a Ψ synthase–FU RNA complex in which the enzyme is clearly covalently attached to the 6-position of the rearranged FU, however, the covalently bound amino acid is the highly conserved Tyr140. This result, in the context of previously reported evidence, provides new insights into the Ψ synthase reaction mechanism.

Huang *et al.* (5) proposed that an aspartate residue (Asp60 in TruA) that is conserved in all known Ψ synthase sequences is involved in catalysis and mutation of the corresponding Asp residues in TruA (5), TruB (39), RluA (39) and RsuA (40) abrogates catalytic activity. Based on the known chemistry of thymidylate synthase and covalent bond formation with the mechanism-based inhibitor FU-tRNA, it was proposed that the conserved Asp initiates and facilitates the reaction by attacking the substrate uridine at C6 (6) (Scheme 1). Based on model chemical counterparts, it was rationalized that this single parsimonious modification to form a covalent 5,6-dihydropyrimidine adduct could (i) increase susceptibility of glycosidic bond cleavage; (ii) provide an axis for 180° rotation of the pyrimidine to juxtapose the C5 position and C1′ of the ribose; and (iii) activate the C5 for reaction with the electrophilic C1′ of the sugar.

In crystal structures of Ψ synthases complexed with FU-containing RNA the bound FU is hydrated at C6 and rearranged to the *C*-glycoside isomer to give (5S, 6R)-5-fluoro-6-hydroxy-Ψ in the substrate/product site (9,11–14). Although the conserved Asp is not covalently attached as an ester to C6 of the rearranged FU, it could be rationalized that cleavage of the ester occurred under conditions of crystallization and/or X-ray irradiation (14). The rearrangement of FU in the native Ψ synthase complexes suggests that chemistries necessary for much of the reaction had already occurred, and lends credence to some aspects of the earlier proposed mechanism. However, as more experimental evidence and information was accumulated, it became clear that the mechanism was more complex.

Biochemical studies of Ψ synthases in complex with FU-containing RNA have contributed to the remainder of our current knowledge of the mechanism. TruA (5,6) and RluA (41) form covalent complexes with FU-containing RNA that can be isolated under denaturing conditions such as SDS- or Urea-PAGE. However, upon heating, the complexes disrupt to non-covalent components. In early studies of TruA, the heat-disrupted adduct yielded an oligonucleotide containing a modified nucleoside that was believed to be 5-fluoro-6-hydroxy-uridine (6-OH-5-FU) (Scheme 2) based on HPLC co-migration with an authentic sample (6). However, a later study (42) indicated that the product was likely a di-nucleotide containing the 6-hydroxy form of the rearranged 6-hydroxy adduct 6-OH-5-FΨ, as observed in crystallographic studies. In contrast, the TruB-FU T-arm stem-loop (TSL) complex could not be isolated by SDS-PAGE (43). Instead, the RNA behaved as a substrate for the enzyme with multiple turnovers to give the oligonucleotide containing the 6-hydroxy-rearranged product (6-OH-5-FΨ). Again, this might be simply explained in context of the proposed mechanism if the Asp-C6 ester (6-Asp-5-FΨ) disrupts rapidly after its formation to prevent its isolation and allow dissociation of a weakly bound oligonucleotide product containing 6-OH-5-FΨ.
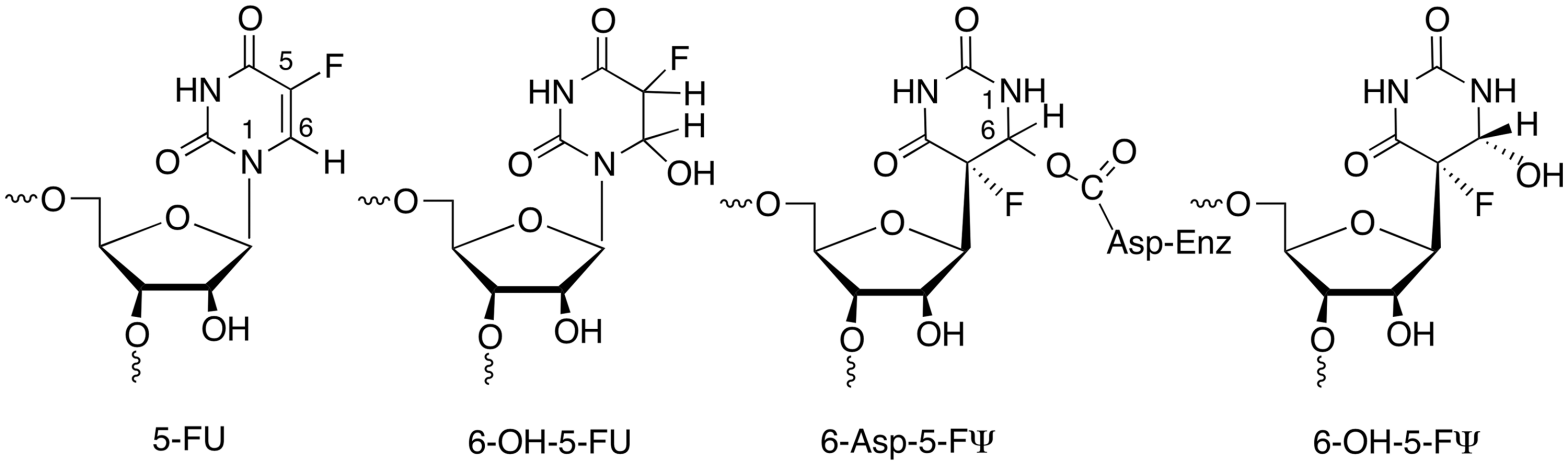

**Scheme 2.** Chemical structures of 5-fluorouridine analogs.

Mueller *et al.* then performed revealing experiments in which formation and/or disruption of Ψ synthase–FU RNA complexes (TruB, RluA and TruA) were performed in H_2_O^18^. They showed that the O^18^ was not incorporated in the expected conserved Asp of the proteins but rather in the 6-hydroxy group of the rearranged FU hydrate (7,41,42). Moreover, they also demonstrated that oxygen incorporation to form the 6-hydrate occurred during the heat disruption of the covalent complex rather than its formation. They convincingly demonstrated that the formation of the 6-hydrate (6-OH-5-FΨ) was not due to a mechanism involving conventional O-acyl bond hydrolysis of the putative Asp-C6 ester (6-Asp-5-FΨ) of a covalent complex. Rather the complex undergoes a simple reversal of the Michael addition from which it was formed and O^18^ incorporation occurs after cleavage of the Asp–FΨ bond.

**Scheme 3.**
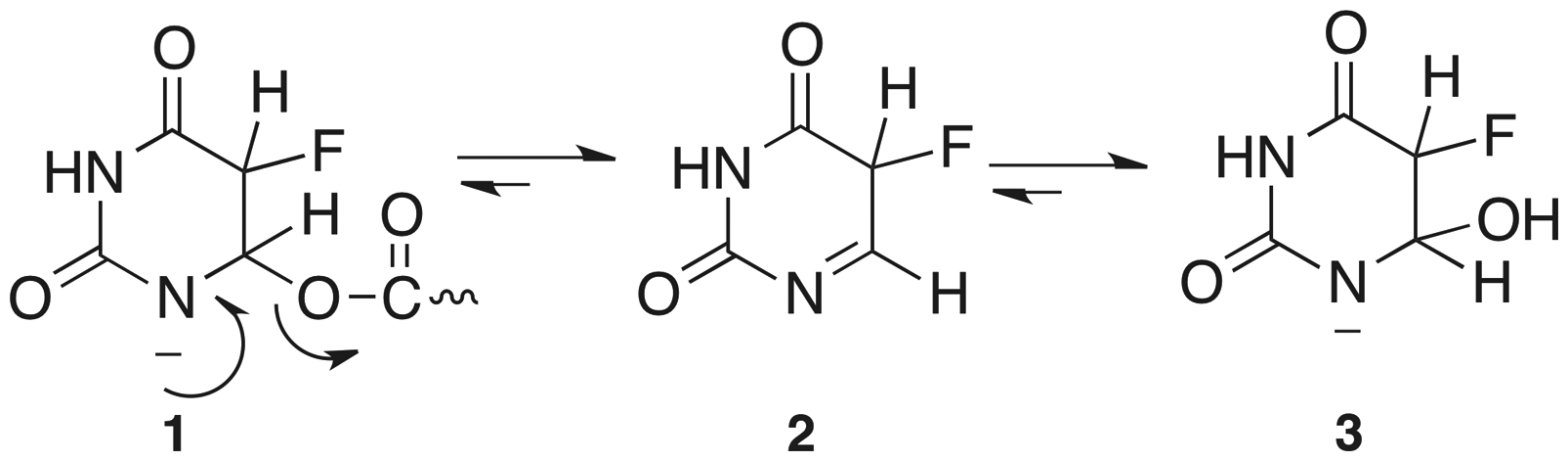
Conversion of 5-F-6-acyloxy-uracil to 5-F-6-hydroxy-uracil.

The chemistry of proposed Asp adducts at C6 of FU have direct analogy to the chemical models 5-F-6-acetoxy and 6-hydroxy-uracil and their 1-substituted counterparts (44–46). When dissolved in water 5-F-6-acetoxy-uracil very rapidly converts to 5-F-6-hydroxy-uracil and then undergoes slower β-elimination to the 5-F pyrimidine. When dissolved in alcohols, 5-F-6-acetoxy-uracil converts to corresponding 6-alkoxy analogs and thus do not undergo O-acyl bond cleavage, but rather O-alkyl cleavage. Corresponding 1-substituted analogs are markedly more stable toward solvolytic reactions, with 1-methyl-5-F-6-acetoxy-uracil undergoing only a few percent reaction per day. The likely mechanism of these reactions involves participation of the N-1 electrons, facilitated by partial or complete ionization of the 1-NH to form a reactive planar intermediate **2** that can undergo reaction with nucleophiles from either face of the pyrimidine ring (Scheme 3). The better leaving group ability of a carboxylate anion versus hydroxide explains the relative reactivities and stabilities of the 6-acyloxy and 6-hydroxy substituents. This chemistry nicely fits the data supporting initial formation of a covalent adduct between Ψ synthase and FU-RNA that ultimately converts to a 6-hydroxy-5-fluorouracil analog. Interestingly, **1** is analogous to an intermediate suggested in the earliest proposal of a mechanism for Ψ synthase when Cys was still a candidate for the nucleophilic catalyst (5).

**Scheme 4.**
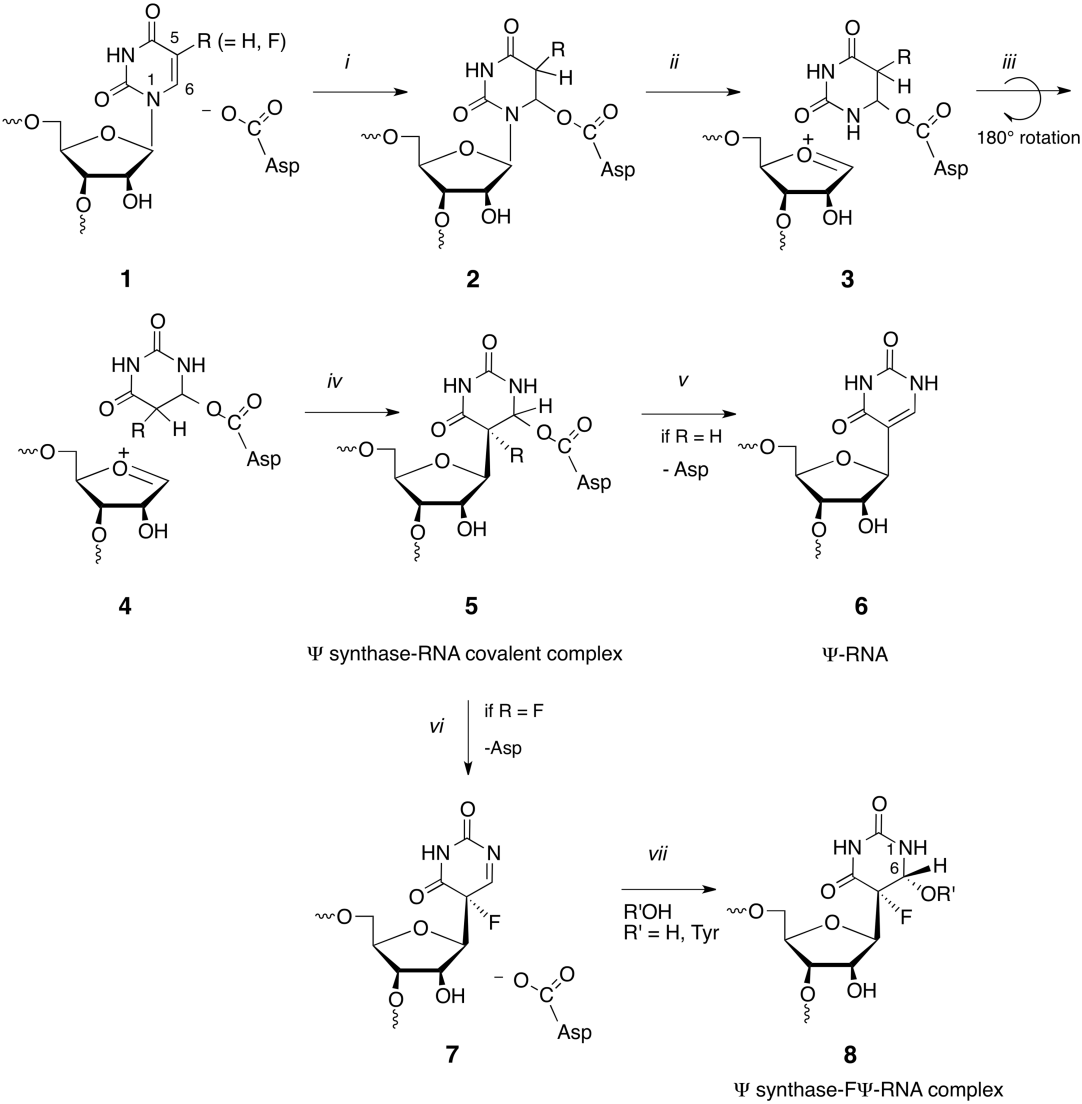
Complete current mechanism of Ψ synthase and its reaction with 5-FU-RNA.

The Michael addition to C6 has precedence in numerous mechanistically related enzymes, which adds credence to the analogous mechanism favored here: these enzymes include thymidylate synthase (47), HmdCMP (48) and HmdUMP (49) transferases, the DNA methyl transferases (50) and the RNA methyl transferases (51). Nevertheless, one should consider whether the alternative acylal mechanism—where catalysis involves initial attack at C1′ of the ribose by Asp—might also account for the reported data. It is generally accepted that the denatured covalent Ψ complex involves covalent adduct formation of the enzyme to C6 of the target base in RNA (6). If this acylal mechanism were operative, there would be no role for the Asp-C6 covalent bond formation in catalysis. That is, an acylal mechanism would involve (i) cleavage of the glycosidic bond by reaction of Asp at C1′ and release of the pyrimidine; (ii) rotation of the substrate U to juxtapose C1′ with C5; (iii) *C*-glycoside formation; and (iv) for no relevant reason, conformational changes of the Asp and covalent bond formation to C6. The latter reaction would occur after reaction completion, and represent an ‘accidental’ event. Therefore the acylal mechanism seems overly complicated and unnecessary.

Scheme 4 shows the Michael reaction mechanism for formation of Ψ (**6**), formation of the Ψ synthase–FU covalent complex (**5**) and the 6-OH-5-FΨ final product (**8**). Here, the conserved Asp initiates the reaction by forming a covalent ester adduct at C6 of the target pyrimidine residue (**2**). This single modification forming a 5,6-dihydropyrimidine adduct could increase susceptibility of glycosidic bond cleavage (ii); provide an axis for 180° rotation of the pyrimidine to juxtapose the C5 position for coupling to the C1′ (iii); and activate the C5 for reaction with the electrophilic C1′ of the sugar (iv). With FU-RNA, C1′–C5 bond formation would provide covalent adduct **5** which would undergo a 1,2-elimination of the leaving group Asp to give highly reactive **7**; hydration to give the observed FΨ 6-hydrate, **8,** which as described above for the models should be significantly more stable than **5**. It can be seen that this mechanism would provide **8** without O-acyl ester cleavage, and hence explain how heat disruption of the covalent complex **5** would give **8** with O^18^ incorporation at the 6-hydroxyl rather than Asp. With substrate uridine a simple β-elimination on **5**, R = H would provide the product Ψ, **6**, R = H.

The RluB–FΨ covalent complex described in the present work is readily accommodated by Tyr addition to **7** shortly after release of Asp60 facilitated by the N-1 electrons to form a planar sp^2^ hybridized C6. Although the phenolic hydroxyl is not a potent nucleophile (pKa ∼10), it is certainly more nucleophilic than water, which does form a FU hydrate in other Ψ synthases. Interestingly, the covalently bound Tyr is on the opposite side of the plane of the FΨ ring as the catalytic Asp and the configuration of the bound Tyr covalent adduct is 5S, 6R—the same configuration as the 6-OH-5-FΨ hydrates found in other Ψ synthase 5-FU-RNA structures; thus, water or Tyr attacks **7** from the same side of the ring.

An accounting of all the proton transfers during pseudouridine formation is shown in the Supplementary Data (Scheme S1). The reaction invokes three acid-base groups, which are needed to accept protons from O2 and O4 and from C5. These acceptors/donors are as yet undefined; waters or backbone amide groups are possible candidates. The active site of the RluB–RNA complex contains three waters that could serve this role. We point out that in all structures of Ψ synthase–RNA complexes thus far reported, the O4 of the substrate pyrimidine is hydrogen bonded to the backbone amide of Asp110, and O2 accepts a hydrogen bond from the main chain N-Hs of residues 196 and/or 197 (RluB numbering).

The fortuitous addition of Tyr140 instead of water to C6 shows that these additions at C6 occur after, and independent of the elimination of the Asp from the covalent RNA adduct. The mechanism proposed here is the most parsimonious explanation of this and all reported mechanistic and structural data on Ψ synthases.

## ACCESSION NUMBERS

Coordinates and structure factors have been deposited in the Protein Data Bank with accession codes 4LAB (apo-RluB) and 4LGT (RluB–RNA complex).

## SUPPLEMENTARY DATA

Supplementary Data are available at NAR Online.

Supplementary Data
