# Supplementary material for: The mechanism of pseudouridine synthases from a covalent complex with RNA, and alternate specificity for U2605 versus U2604 between close homologs
Source: Nucleic Acids Res. 2013 Nov 7;42(3):2037–48. doi: 10.1093/nar/gkt1050 (PMC3919597; doi:10.1093/nar/gkt1050)
Supplement: Supplementary Data [file supp_42_3_2037__index.html]

The mechanism of pseudouridine synthases from a covalent complex with RNA, and alternate specificity for U2605 versus U2604 between close homologs — The mechanism of pseudouridine synthases from a covalent complex with RNA, and alternate specificity for U2605 versus U2604 between close homologs — Supplementary Data 

# The mechanism of pseudouridine synthases from a covalent complex with RNA, and alternate specificity for U2605 versus U2604 between close homologs

## Supplementary Data

files

**Files in this Data Supplement:**

- Supplementary Data - pdf file
