# Supplementary material for: The mechanism of pseudouridine synthases from a covalent complex with RNA, and alternate specificity for U2605 versus U2604 between close homologs
Source: Nucleic Acids Res. 2013 Nov 7;42(3):2037–48. doi: 10.1093/nar/gkt1050 (PMC3919597; doi:10.1093/nar/gkt1050)
Supplement: Supplementary Data [file supp_gkt1050_nar-01951-r-2013-File011.pdf]

Supplementary Data

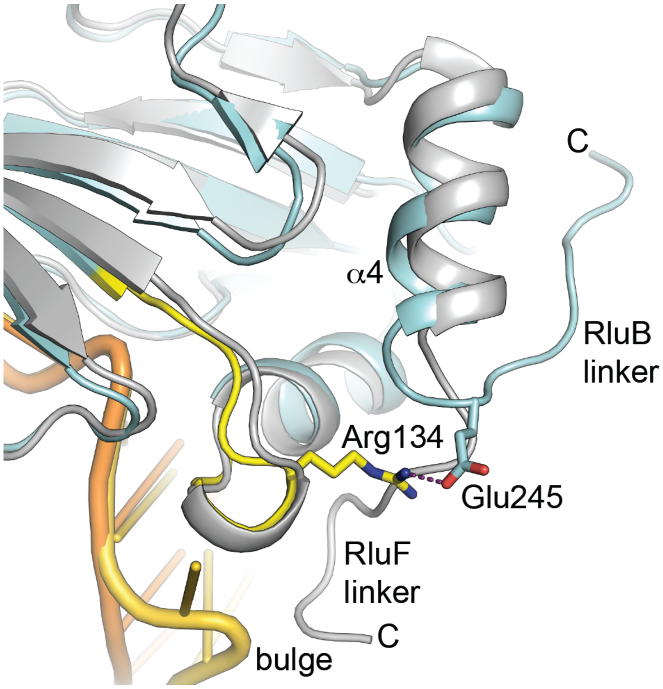

Figure S1. Overlay of the RluB and RluF RNA complexes in the vicinity of the bulge. RluB and its RNA substrate are in blue-green and gold, respectively. RluF and its RNA substrate are grey and orange, respectively. The α1-β4 linking residues 131-138 in RluB are highlighted in yellow. In RluB Arg134 hydrogen bonds to Glu245, reinforcing a hairpin turn that directs the chain away from the bulge-binding site.

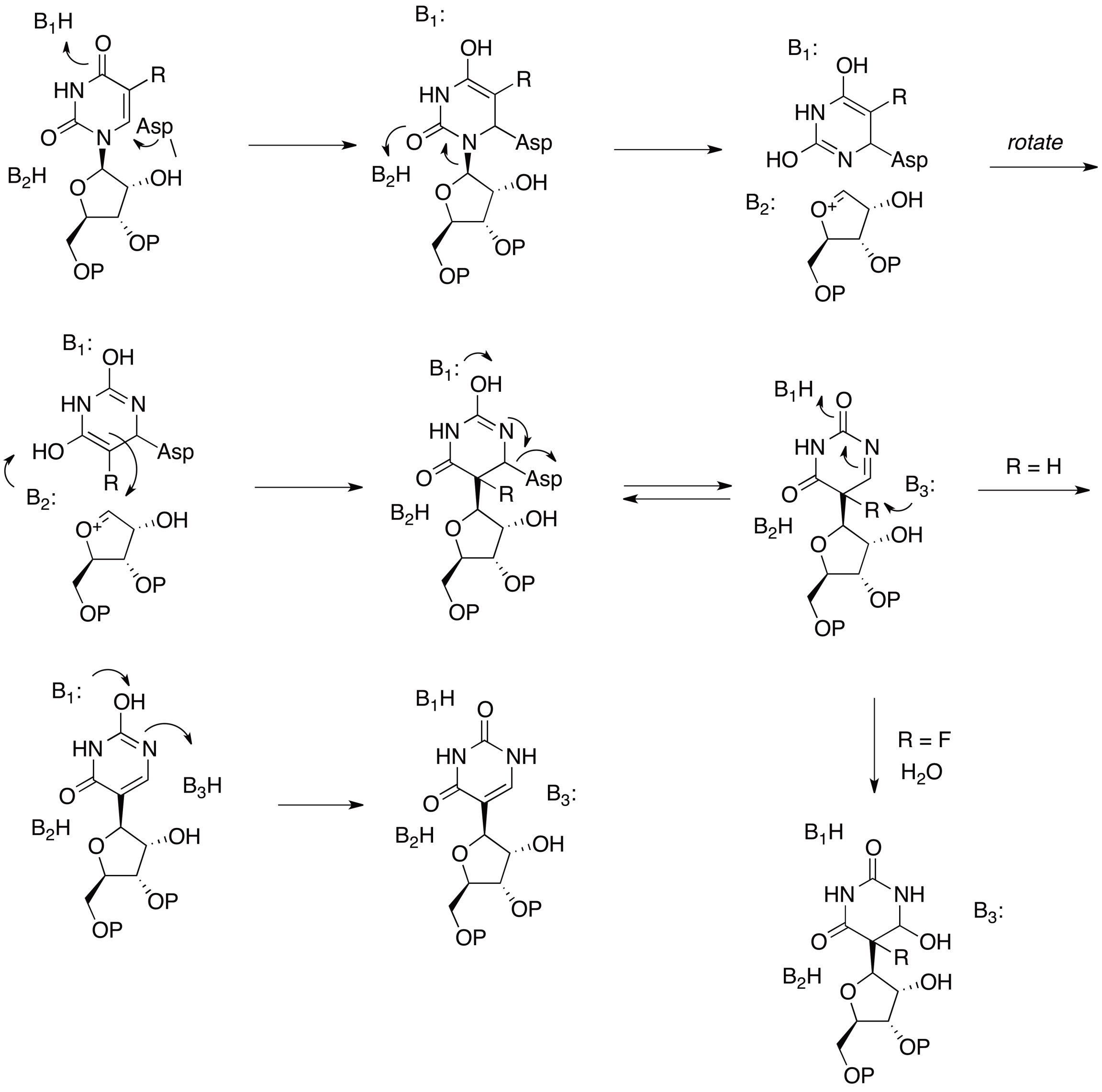

Scheme S1. Proton transfers during Ψ formation
